# Impacts of hempseed and multicarbohydrase on growth performance, digestibility, intestinal morphology and microbial population of broilers

**DOI:** 10.1016/j.psj.2026.107993

**Published:** 2026-10-18

**Authors:** Mojtaba Moaddab Gaskarei, Farid Shariatmadari, Mohammad Amir Karimi Torshizi, Hamed Ahmadi

**Affiliations:** Department of Poultry Science, Faculty of Agriculture, Tarbiat Modares University, Tehran, 14115-338, Islamic Republic of Iran

**Keywords:** Hempseed, Multicarbohydrase, Protein source, Broiler

## Abstract

This study evaluated the effects of hempseed (H) inclusion and multicarbohydrase (MC; xylanase and β-glucanase) supplementation on growth performance, apparent ileal digestibility (AID), intestinal morphology, and selected ileal bacterial populations in broilers. A total of 768 one-day-old male Ross 308 chicks received a common starter diet to d 7 and, from d 8 (mean initial BW 164 g, P > 0.05 among treatments) to d 28, were assigned to a 4 × 2 factorial arrangement of four hempseed levels (0, 8, 16, and 24%) and two MC levels (0 or 0.104 g/kg). No hempseed × MC interaction was observed for growth performance (P > 0.05). Hempseed increased body wight (BW; P < 0.001) and BW gain (P = 0.006) and reduced feed conversion ratio (FCR; P = 0.036), while MC improved BW gain (P = 0.041) and FCR (P = 0.011). Hempseed increased AID of crude protein (CP), ether extract (EE), and gross energy (GE) and decreased AID of dry matter, neutral detergent fiber (NDF), and acid detergent fiber (ADF; P < 0.001). MC increased AID of dry matter, organic matter, CP, NDF, and GE (P ≤ 0.004). Significant interactions were found for AID of EE, CP, ADF, and GE (P ≤ 0.03): MC increased AID of CP only in the control diet and AID of GE at 0 and 8% hempseed, but reduced AID of EE at 24% hempseed. Hempseed increased villus height (VH) and villus height to crypt depth (VH:CD) ratio in all segments (P < 0.001), and MC reduced crypt depth (CD) in the duodenum and jejunum (P ≤ 0.01). Interactions for duodenal VH (P = 0.043) and VH:CD ratio (P = 0.033) and jejunal VH:CD ratio (P = 0.029) indicated that MC increased the duodenal VH and VH:CD at 0 and 8% hempseed but not at 16 and 24%. Hempseed and MC modestly increased ileal *Lactobacillus* counts (P < 0.001) and reduced total aerobic (P < 0.001) and coliform counts (P ≤ 0.032), with interactions for total aerobic bacteria (P < 0.001) and *Lactobacillus* (P = 0.009). In conclusion, up to 24% hempseed supported growth performance and was associated with higher AID of CP, EE, and GE and improved villus morphology. The response to MC depended on hempseed level, with its effects on digestibility and duodenal morphology most evident at 0 and 8% hempseed. These findings are limited by the short experimental period (28 d) and by culture-based enumeration of only three bacterial groups.

## Introduction

The escalating cost and limited availability of conventional protein sources have intensified the search for sustainable and alternative feed ingredients in poultry nutrition. Hempseed (Cannabis sativa) has emerged as a valuable alternative protein source in poultry nutrition due to its favorable amino acid profile and rich content of essential fatty acids, particularly omega-3 and omega-6 (Shariatmadari, 2023). Hempseed protein is mainly composed of edestin and contains all essential amino acids, with a relatively high proportion of essential amino acids and high levels of arginine and glutamic acid (Wang et al., 2022; Callaway, 2004). The proportion of essential amino acids relative to total amino acids in hempseed protein has also been reported to be higher than that of soybean protein (Shariatmadari, 2023). These nutritional characteristics make hempseed a promising substitute for conventional protein sources used in broiler diets. Previous studies have reported beneficial effects of dietary hempseed on body weight gain and feed efficiency in broilers, as well as improvements in the fatty acid profile of broiler meat through increased omega-3 content, which may enhance its nutritional value for human consumption (Kanbur, 2022; Tufarelli et al., 2023; Serrapica et al., 2025). Thus, available evidence indicates that hempseed has considerable nutritional potential as an alternative feed ingredient for broilers.

Despite these advantages, the relatively high fiber content of hempseed, particularly its non-starch polysaccharide **(NSP)** fraction, may limit its nutritional utilization in poultry diets. NSPs are considered anti-nutritional factors because they can increase digesta viscosity, interfere with nutrient absorption, and consequently impair growth performance in poultry (Hetland et al., 2004). Therefore, the NSP content of hempseed-containing diets may limit nutrient utilization, highlighting the potential role of exogenous carbohydrases in improving nutrient digestibility (Shahtalab et al., 2026). Carbohydrases such as xylanase and β-glucanase are widely used in poultry nutrition to degrade NSPs, reduce digesta viscosity, and enhance nutrient availability (Adeola and Cowieson, 2011). In addition, previous research has shown that supplementation with carbohydrases, containing arabinofuranosidase, improves energy and protein utilization, nutrient digestibility, growth performance, and intestinal morphology in broilers, as evidenced by increased villus height and optimized crypt depth, which are indicators of enhanced absorptive capacity (Cozannet et al., 2017; Saleh et al., 2019).

Although the nutritional value of hempseed and the potential benefits of carbohydrase supplementation have been investigated separately, the combined response to increasing levels of hempseed with and without multicarbohydrase **(MC)** complexes (xylanase and β-glucanase) has received comparatively limited investigation, particularly with respect to nutrient digestibility, intestinal morphology, microbial populations, and growth performance. More specifically, it remains important to determine whether MC supplementation can improve the utilization of nutrients in hempseed-containing diets and whether the magnitude of its effects varies with the level of hempseed inclusion. Addressing this question is particularly relevant because increasing hempseed inclusion may increase the contribution of its fiber and NSP fraction to the diet, potentially creating greater opportunity for NSP-degrading enzymes to influence nutrient utilization and intestinal responses.

Therefore, the objective of the present study was to evaluate the effects of four levels of hempseed inclusion with or without MC supplementation on growth performance, intestinal morphology, intestinal microbial populations, and ileal apparent digestibility of broiler chickens. We hypothesized that increasing hempseed inclusion would influence nutrient utilization and intestinal characteristics and that MC supplementation would enhance these responses in hempseed-containing diets, with its effects potentially becoming more evident at higher levels of hempseed inclusion.

## Materials and methods

### Ethical statement

The experimental procedures for animal trials were approved by the Tarbiat Modares University Animal Ethics Committee (IR.MODARES.REC.1400.032) and complied with Iranian guidelines for the care and use of animals in scientific research.

### Birds and husbandry

A total of 768 one-day-old male Ross 308 broiler chicks were obtained from a commercial hatchery and randomly allocated to 64 floor pens (2.0 × 1.0 × 0.8 m), with 12 birds per pen. The 64 pens were randomly assigned to the eight experimental treatments, with eight replicate pens per treatment, and treatments were distributed across the experimental room in a balanced manner to minimize potential location and environmental effects. All pens were equipped with nipple drinkers and a trough feeder and were bedded with fresh wood shavings at a uniform depth of 4 cm. The room temperature was initially maintained at 32 °C and gradually reduced by 2.5 °C per week until reaching 22 °C. Lighting was provided continuously for the first 24 h after placement. Thereafter, the dark period gradually increased to 6 h by day 7. Feed and water were provided *ad libitum* throughout the experiment. All diets were offered in mash form.

### Experimental design and treatments

Birds received a common commercial starter diet from day 0–7. From day 8 until the end of the experiment (day 28), a 4 × 2 factorial arrangement was employed, consisting of four hempseed inclusion levels (0, 8, 16, and 24%) and two MC supplementation levels (without or with MC). This design resulted in eight dietary treatments: 0% hempseed without MC (**H0E0**; control), 8% hempseed without MC (**H8E0**), 16% hempseed without MC (**H16E0**), 24% hempseed without MC (**H24E0**), 0% hempseed with MC (**H0E1**), 8% hempseed with MC (**H8E1**), 16% hempseed with MC (**H16E1**), and 24% hempseed with MC (**H24E1**). Each treatment was replicated in eight pens, with 12 birds per pen**.** To determine apparent ileal digestibility (**AID**), titanium dioxide (**TiO₂**) was included in the experimental diets at 5 g/kg feed during the final five days of the trial (days 24–28) as an indigestible marker.

### Diet formulation and hempseed characterization

All diets were formulated to be isoenergetic and isonitrogenous and to meet or exceed the nutrient requirements of Ross 308 broiler chickens (Aviagen, 2022). Ingredient composition and calculated nutrient contents of the experimental diets are presented in Table 1. Chemical analyses were performed according to AOAC (2000) procedures. The chemical composition of the ground hempseed used in the study is presented in Table 2.

**Table 1:** Feed ingredients of experimental treatments.

Ingredients (g kg^–1^)	1–7 day	8–28 day							
		H0E0	H0E1	H8E0	H8E1	H16E0	H16E1	H24E0	H24E1
Corn	529.00	523.05	522.95	499.54	499.43	476.02	475.91	452.50	452.39
Soybean meal 44%	400.00	393.11	393.11	350.12	350.12	307.13	307.13	264.14	264.14
Soybean Oil	21.50	42.47	42.47	28.34	28.34	14.21	14.21	0.07	0.07
Calcium carbonate	16.10	14.00	14.00	14.38	14.38	14.76	14.76	15.15	15.15
Salt	4.00	3.83	3.83	3.86	3.86	3.88	3.88	3.90	3.90
Vitamin/mineral premix a	5.00	5.00	5.00	5.00	5.00	5.00	5.00	5.00	5.00
Monocalcium Phosphate	17.00	14.37	14.37	13.45	13.45	12.54	12.54	11.62	11.62
L-Lysine	2.50	0.91	0.91	1.47	1.47	2.03	2.03	2.59	2.59
Threonine	1.20	0.33	0.33	0.65	0.65	0.96	0.96	1.28	1.28
DL-Methionine	3.70	2.93	2.93	3.21	3.21	3.48	3.48	3.76	3.76
Hempseed	0.00	0.00	0.00	80.00	80.00	160.00	160.00	240.00	240.00
Multicarbohydrase	0.00	0.00	0.104	0.00	0.104	0.00	0.104	0.00	0.104
Total	1000	1000	1000	1000	1000	1000	1000	1000	1000
ME (Kcal/kg)	2890	3030	3030	3030	3030	3030	3030	3030	3030
CP %	22.11	21.50	21.50	21.50	21.50	21.50	21.50	21.50	21.50
Lysine (%)	1.38	1.24	1.24	1.24	1.24	1.24	1.24	1.24	1.24
Methionine + Cysteine (%)	1.03	0.95	0.95	0.95	0.95	0.95	0.95	0.95	0.95
Threonine (%)	0.94	0.84	0.84	0.84	0.84	0.84	0.84	0.84	0.84
Available P (%)	0.50	0.44	0.44	0.44	0.44	0.44	0.44	0.44	0.44
Ca (%)	1.00	0.87	0.87	0.87	0.87	0.87	0.87	0.87	0.87
Na (%)	0.17	0.16	0.16	0.16	0.16	0.16	0.16	0.16	0.16
Ca/P availability	2.00	2.00	2.00	2.00	2.00	2.00	2.00	2.00	2.00
Nutrient composition									
Crude protein (%)		21.65	21.57	21.95	21.61	21.78	21.88	21.45	21.74
Neutral detergent fiber (%)		28.92	30.82	31.90	32.84	33.84	35.34	36.59	36.36
Acid detergent fiber (%)		18.33	19.02	19.96	19.78	22.30	21.92	23.88	23.97

a Each kg of vitamin and mineral premix contained: Vitamin A 4000000 IU, vitamin E 26000 IU, vitamin D3 1800000 vitamin B6 1280 mg, Folic acid 760 mg, Biotin 72 mg, vitamin B12 6.8 mg, choline chloride 320000 mg and antioxidant 1000 mg, Fe, 8000 mg, Mn, 48000 mg, Cu, 6400 mg, I, 500 mg, Zn, 44000 mg, Se, 120 mg.

**Table 2:** Nutrient composition of hempseed.

Hempseed composition		
Dry matter (%)	94.62	
Gross energy (Kcal/kg)	5925	
Crude protein (%)	24.7	
Ether extract (%)	30.5	
Ash (%)	5.37	
Crude fiber (%)	29.6	
Neutral detergent fiber (%)	32.4	
Acid detergent fiber (%)	22.1	
Total Lysine (%) Total Methionine (%) Total Threonine (%)	1.02 0.43 0.62	

### Multicarbohydrase preparation and activity assay

Two non-starch polysaccharide-degrading enzymes were obtained from AB Vista (Wageningen, The Netherlands): Econase XT (endo-1,4-β-xylanase; minimum activity of 4,000,000 BXU/g) and Econase GT 200 (endo-1,3(4)-β-glucanase; minimum activity of 200,000 BU/g). The multicarbohydrase treatment consisted of a blend containing 4 g/ton of Econase XT and 100 g/ton of Econase GT 200. The activities of the commercial enzyme preparations were verified prior to diet formulation. Xylanase activity was measured by quantifying the release of reducing sugars from 1% birchwood xylan in 0.1 M sodium acetate buffer (pH 5.5) using the dinitrosalicylic acid (**DNS**) method. Following incubation at 37 °C for 30 min, DNS reagent was added and the mixture was boiled for 5 min. Absorbance was measured at 540 nm using a microplate reader (Dhaver et al., 2022). β-Glucanase activity was determined using 1.5% barley β-glucan as substrate under the same DNS-based procedure. The enzyme was incubated at 40 °C for 20 min in 100 mM acetate buffer (pH 5.0), and reducing sugars released during hydrolysis were quantified by measuring absorbance at 530 nm (König et al., 2002). One unit (**U**) of β-glucanase activity was defined as the amount of enzyme required to release 1 μmol of reducing sugars per minute under the assay conditions. Enzyme activity was expressed as U/g. All assays were performed in triplicate, and only results with a relative standard deviation (**RSD**) below 5% were accepted.

### Growth performance

Growth performance was evaluated using final body weight (**BW**), body weight gain (**BWG**), feed intake (**FI**), and feed conversion ratio (**FCR**). BW was recorded at placement, immediately before the introduction of the experimental diets (8 d of age), and at the end of the trial (28 d of age). The experimental diets were offered from day 8–28, and BWG was calculated for this period. Feed intake was calculated on a pen basis as the difference between feed offered and residual feed. Feed conversion ratio was obtained for each pen as the ratio of FI to BWG. Performance data were corrected for mortality when a bird died during the experimental period. Mortality was recorded throughout the trial and was 3.12, 2.08, 1.04, 2.08, 1.04, 3.12, 1.04, and 4.16% for H0E0, H0E1, H8E0, H8E1, H16E0, H16E1, H24E0, and H24E1, respectively, with an overall mortality rate of 2.21%.

### Ileal microbial population

On day 28, two birds per pen were selected with body weights close to the replicate mean group average and slaughtered for microbiological analysis. The ileum (from Meckel’s diverticulum to 5 cm before the ileocecal junction) was excised, and about 1 g of homogenized digesta was collected (samples of each pen were pooled). Samples were serially diluted in sterile saline (1:9 w/v), and bacterial counts were determined using the droplet method. Plate Count Agar (Merck, Darmstadt, Germany), MacConkey Agar (Himedia Laboratories, Mumbai, India), and Man–Rogosa–Sharpe Agar (Merck, Darmstadt, Germany) were used to enumerate total aerobic bacteria, *coliform* bacteria, and *Lactobacillus* spp., respectively. Plates were incubated aerobically at 37 °C for 24 h before colony-forming units (**CFU**) were counted. Microbial counts were transformed and reported as log10 CFU/g of ileal digesta.

### Intestinal morphology assessment

Duodenum, jejunum, and ileum segments (2 cm) were collected from one bird per pen. Samples were immediately preserved in 10% buffered formalin solution for histological evaluation. After fixation, tissues were dehydrated through a graded ethanol series (70% to absolute alcohol), cleared with xylene, and embedded in paraffin wax. Sections of 5 µm thickness were prepared. The sections were then deparaffinized and stained with hematoxylin and eosin as well as Alcian blue. Morphometric analysis was performed using a light microscope (Standard 20, Carl Zeiss, Göttingen, Germany) equipped with imaging software (Dino-Capture 2.0; Taiwan) and an ocular micrometer. Villus height (**VH**), crypt depth (**CD**), villus width (**VW**), villus surface area (**VSA**), and goblet cell number (**GN**) were measured. The villus height to crypt depth ratio (**VH:CD**) was also calculated. For each sample, five well-oriented villi and their associated crypts were measured, and mean values were used for statistical analysis.

### Apparent ileal digestibility

On day 28, AID was assessed by selecting two birds per replicate, with body weights close to the replicate mean group average. Birds were euthanized by cervical dislocation, and ileal digesta were collected. The ileal (distal part of the ileum, 10 cm proximal to the ileocecal junction) contents were gently collected into labeled containers and pooled by replicate. Thus, the pooled ileal digesta sample from the two birds represented one experimental unit per replicate (pen) for AID analysis. Samples were immediately frozen at –20 °C and later oven-dried at 55 °C until a constant weight was reached. Dry matter (**DM**), organic matter (**OM**), crude protein (**CP**), crude fiber (**CF**), and ether extract (**EE**) were analyzed following AOAC methods (2000). Neutral detergent fiber (**NDF**) and acid detergent fiber (**ADF**) were determined according to the method of Van Soest et al. (1991). Gross energy (**GE**) values of the ileal content were measured using an adiabatic calorimeter (Gallenkamp Autobomb; UK). Titanium dioxide concentration in diets and ileal digesta was determined according to the method of Short et al. (1996). Apparent ileal digestibility coefficients were calculated using titanium dioxide as an indigestible marker according to the following equation:

AID (%) = 100 − [(TiO₂ diet / TiO₂ digesta) × (Nutrient digesta / Nutrient diet) × 100]

### Statistical analysis

Data were analyzed according to a 4 × 2 factorial arrangement using the General Linear Model (**GLM**) procedure of SAS software (version 9.4; SAS Institute Inc., Cary, NC, USA). The statistical model included the main effects of hempseed (**H**), enzyme (**E**), and their interaction (**H** **×** **E**), which was tested within the ANOVA model. The pen served as the experimental unit for all measurements, including growth performance, apparent ileal digestibility, microbial population, and intestinal morphology. When significant differences among treatments were detected (P < 0.05), means were separated using Tukey’s multiple range test. Orthogonal polynomial contrasts were used to evaluate linear and quadratic responses to increasing levels of dietary hempseed. Data are presented as treatment means, with the pooled standard error of the mean (**SEM**) reported separately in the tables.

## Results

### Enzyme activity

β-Glucan content of the experimental diets was approximately 1%. Enzyme activities were confirmed prior to diet formulation using in vitro assays. Xylanase activity was 4,520,000, 4,380,000, and 4,495,000 BXU/g (mean: 4,465,000 BXU/g; RSD: 1.67%). β-Glucanase activity was 223,000, 236,500, and 218,000 BU/g (mean: 225,833 BU/g; RSD: 4.24%). Based on the measured enzyme activities and their inclusion rates, the multicarbohydrase supplementation provided approximately 17,860 BXU/kg of xylanase and 22,583 BU/kg of β-glucanase in the complete feed.

### Growth performance

Growth performance data are illustrated in Table 3. Hempseed × MC interaction showed no significant effect on BW, BWG, FI, or FCR (P > 0.05), indicating that the effects of hempseed inclusion and enzyme supplementation were not statistically dependent on each other.

**Table 3:** Influence of hempseed inclusion and enzyme supplementation on growth performance in broilers at 28 d of age.

Treatments	Body weight (g)		Body weight gain (g)	Feed intake (g)	Feed conversion ratio
	Day 8	Day 28			
H0E0	164.79	1200	1051	1373	1.308
H0E1	160.18	1223	1066	1351	1.268
H8E0	163.33	1245	1086	1377	1.268
H8E1	163.12	1279	1114	1385	1.243
H16E0	165.79	1277	1091	1389	1.274
H16E1	163.23	1269	1117	1361	1.219
H24E0	165.83	1247	1080	1345	1.245
H24E1	165.62	1250	1091	1355	1.242
SEM	1.84	10.82	13.64	18.97	0.016
Interaction (Hempseed × Enzyme)					
p-value	0.579	0.211	0.897	0.662	0.444
Main effects					
Inclusion level of hempseed					
H0	162.48	1212^b^	1058^b^	1362	1.287^a^
H8	163.23	1262^a^	1100^a^	1381	1.255^ab^
H16	164.51	1273^a^	1104^a^	1375	1.246^ab^
H24	165.72	1248^a^	1085^ab^	1350	1.243^b^
SEM	1.30	7.65	9.64	13.41	0.011
p-value	0.316	<0.001	0.006	0.374	0.036
Enzyme					
E0	164.93	1242	1077^b^	1371	1.274^a^
E1	163.04	1255	1097^a^	1363	1.243^b^
SEM	0.92	5.41	6.82	9.48	0.008
p-value	0.151	0.09	0.041	0.562	0.011
Orthogonal polynomial contrast					
L	-	0.006	0.181	0.451	0.028
Q	-	0.008	0.141	0.278	0.764

Means in the same column with different superscripts (^abc^) are significantly different.

SEM: pooled standard error of the mean.

H0: 0% hempseed, H8: 8% hempseed, H16: 16% hempseed, H24: 24% hempseed. E0: without enzyme, E1: 0.104 g/kg enzyme.

L: linear effect of inclusion level of hempseed, Q: quadratic effect of inclusion level of hempseed.

On the other hand, BW increased with hempseed inclusion (P < 0.001), although there is no significant difference between H8, H16, and H24. and at 16% hempseed exhibited higher weight. Both linear (P = 0.006) and quadratic (P = 0.008) responses of inclusion level of hempseed were detected for final BW. Hempseed inclusion could also increase BWG (P = 0.006), with an upward trend from 0% to 16% hempseed; however, neither the linear nor quadratic inclusion level of hempseed contrast for BWG was significant (P > 0.05). Feed intake was not significantly affected by hempseed inclusion (P = 0.374) and MC supplementation (P = 0.562). Inclusion of hempseed improved FCR (P = 0.036), with a decreasing value from 0% to 24% hempseed.

### Ileal microbial population

The effects of hempseed inclusion and MC supplementation on ileal bacterial populations at 28 d are presented in Table 4. Significant H × MC interactions were observed for total aerobic bacteria (P < 0.001) and *Lactobacillus* spp. (P = 0.009), whereas no interaction was detected for coliform bacteria (P = 0.335). The interaction for total aerobic bacteria reflected a variable numerical response to MC supplementation across hempseed inclusion levels, with the greatest numerical reduction observed at 8% hempseed. For *Lactobacillus* spp., MC supplementation numerically increased counts across hempseed levels, although the magnitude of this response varied among hempseed levels. Total aerobic bacteria, *Lactobacillus* spp., and coliform bacteria were influenced by hempseed inclusion as a main effect (P ≤ 0.009). Significant linear and quadratic responses to increasing hempseed inclusion were observed for all three bacterial populations (P ≤ 0.048). MC supplementation decreased total aerobic and coliform bacteria and increased *Lactobacillus* spp. (P ≤ 0.032).

**Table 4:** Influence of hempseed inclusion and enzyme supplementation on intestine microbial population (log_10_ CFU g^−1^) in broilers at 28 d of age.

Treatment	Total aerobic bacteria	*Lactobacillus* spp.	*coliform* bacteria
H0E0	8.252^a^	8.103^d^	4.275
H0E1	8.185^ab^	8.109^d^	4.166
H8E0	8.139^bc^	8.130^d^	3.959
H8E1	7.776^e^	8.133^d^	3.606
H16E0	8.103^bc^	8.213^bc^	3.893
H16E1	7.891^d^	8.284^a^	3.768
H24E0	8.176^ab^	8.192^c^	3.911
H24E1	8.048^c^	8.233^b^	3.894
SEM	0.019	0.007	0.091
Interaction (Hempseed × Enzyme)			
p-value	<0.001	0.009	0.335
Main effects			
Inclusion level of hempseed			
H0	8.218^a^	8.105^d^	4.221^a^
H8	7.958^c^	8.131^c^	3.783^b^
H16	7.996^c^	8.249^a^	3.831^b^
H24	8.111^b^	8.212^b^	3.903^b^
SEM	0.013	0.005	0.064
p-value	<0.001	<0.001	0.009
Enzyme			
E0	8.167^a^	8.159^b^	4.009^a^
E1	7.975^b^	8.189^a^	3.858^b^
SEM	0.009	0.004	0.046
p-value	<0.001	<0.001	0.032
Orthogonal polynomial contrast			
L	0.001	<0.001	0.006
Q	<0.001	0.007	0.048

Means in the same column with different superscripts (^abc^) are significantly different.

SEM: pooled standard error of the mean.

H0: 0% hempseed, H8: 8% hempseed, H16: 16% hempseed, H24: 24% hempseed. E0: without enzyme, E1: 0.104 g/kg enzyme.

L: linear effect of inclusion level of hempseed, Q: quadratic effect of inclusion level of hempseed.

### Intestinal morphology assessment

The morphology of the duodenum, jejunum, and ileum affected by hempseed inclusion and MC supplementation is shown in Table 5, Table 6, Table 7. In the duodenum, significant H × MC interactions were observed for VH (P = 0.043) and VH:CD (P = 0.033). These interactions reflected variation in the numerical response to MC supplementation across hempseed inclusion levels. In the jejunum, the hempseed × MC interaction was detected as significant only for VH:CD (P = 0.029). No significant H × MC interactions were detected for any of the measured ileal characteristics (P > 0.05).

**Table 5:** Influence of hempseed inclusion and enzyme supplementation on duodenum morphology in broilers at 28 d of age.

Treatment	VH (µm)	CD (µm)	VW (µm)	GN (100 µm)	VH:CD (µm)	VSA (mm^2^)
H0E0	1585.20^c^	308.02	89.58	12.50	5.158^e^	0.445
H0E1	1605.55^c^	286.78	83.75	12.83	5.600^cd^	0.422
H8E0	1633.85^bc^	297.35	96.43	13.50	5.510^de^	0.494
H8E1	1721.80^a^	283.83	96.70	13.83	6.075^bc^	0.522
H16E0	1726.45^a^	264.43	92.38	14.33	6.550^ab^	0.500
H16E1	1747.90^a^	254.18	100.48	14.66	6.883^a^	0.552
H24E0	1707.36^ab^	272.35	93.48	14.16	6.280^b^	0.501
H24E1	1691.86^ab^	277.27	93.67	14.83	6.108^bc^	0.497
SEM	17.74	5.30	2.53	0.32	0.127	0.014
Interaction (Hempseed × Enzyme)						
p-value	0.043	0.11	0.069	0.93	0.033	0.072
Main effects						
Inclusion level of hempseed						
H0	1595.37^c^	297.40^a^	86.67^b^	12.66^b^	5.379^d^	0.434^b^
H8	1677.82^b^	290.59^a^	96.56^a^	13.66^a^	5.793^c^	0.507^a^
H16	1737.17^a^	259.31^c^	96.43^a^	14.50^a^	6.716^a^	0.526^a^
H24	1699.62^ab^	274.81^b^	93.57^a^	14.50^a^	6.194^b^	0.499^a^
SEM	12.55	3.75	1.79	0.22	0.090	0.010
p-value	<0.001	<0.001	0.009	<0.001	<0.001	<0.001
Enzyme						
E0	1663.21^b^	285.54^a^	92.97	13.62	5.874^b^	0.485
E1	1691.77^a^	275.51^b^	93.65	14.04	6.166^a^	0.498
SEM	8.87	2.65	1.26	0.16	0.063	0.007
p-value	0.028	0.01	0.706	0.075	0.002	0.218
Orthogonal polynomial contrast						
L	<0.001	<0.001	0.55	0.001	<0.001	0.02
Q	0.08	0.13	0.32	0.11	0.05	0.13

VH: villus height, CD: crypt depth, VW: villus width, GN: goblet cell count, VH:CD: villus height/crypt depth, VSA: villus surface area.

Means in the same column with different superscripts (^abc^) are significantly different.

SEM: pooled standard error of the mean.

H0: 0% hempseed, H8: 8% hempseed, H16: 16% hempseed, H24: 24% hempseed. E0: without enzyme, E1: 0.104 g/kg enzyme.

L: linear effect of inclusion level of hempseed, Q: quadratic effect of inclusion level of hempseed.

**Table 6:** Influence of hempseed inclusion and enzyme supplementation on jejunum morphology in broilers at 28 d of age.

Treatment	VH (µm)	CD (µm)	VW (µm)	GN (100 µm)	VH:CD (µm)	VSA (mm^2^)
H0E0	861.85	275.18	88.03	12.83	3.14^c^	0.238
H0E1	877.78	245.46	87.85	13.00	3.59^bc^	0.242
H8E0	903.55	243.11	92.00	13.50	3.72^ab^	0.261
H8E1	896.50	229.00	91.86	13.83	3.93^ab^	0.258
H16E0	980.30	237.83	88.28	14.00	4.14^a^	0.272
H16E1	920.90	236.38	89.80	14.50	3.91^ab^	0.259
H24E0	995.25	237.00	92.90	14.83	4.24^a^	0.289
H24E1	938.43	224.63	88.20	14.66	4.18^a^	0.258
SEM	19.19	5.98	3.22	0.53	0.11	0.011
Interaction (Hempseed × Enzyme)						
p-value	0.146	0.144	0.808	0.934	0.029	0.437
Main effects						
Inclusion level of hempseed						
H0	869.81^c^	260.32^a^	87.94	12.92^b^	3.36^c^	0.240^b^
H8	900.02^bc^	236.06^b^	91.93	13.67^ab^	3.82^b^	0.260^ab^
H16	950.60^ab^	237.11^b^	89.04	14.25^ab^	4.02^ab^	0.265^ab^
H24	966.84^a^	230.48^b^	90.46	14.75^a^	4.21^a^	0.274^a^
SEM	13.57	4.23	2.27	0.37	0.08	0.007
p-value	<0.0001	<0.0001	0.632	0.009	<0.0001	0.027
Enzyme						
E0	935.23	248.16^a^	90.26	13.79	3.81	0.265
E1	908.40	233.87^b^	89.43	14.00	3.90	0.254
SEM	9.59	2.99	1.61	0.26	0.05	0.005
p-value	0.055	0.001	0.716	0.585	0.266	0.175
Orthogonal polynomial contrast						
L	<0.0001	0.0006	0.436	0.009	<0.0001	0.007
Q	0.518	0.032	0.935	0.871	0.046	0.825

VH: villus height, CD: crypt depth, VW: villus width, GN: goblet cell count, VH:CD: villus height/crypt depth, VSA: villus surface area.

Means in the same column with different superscripts (^abc^) are significantly different.

SEM: pooled standard error of the mean.

H0: 0% hempseed, H8: 8% hempseed, H16: 16% hempseed, H24: 24% hempseed. E0: without enzyme, E1: 0.104 g/kg enzyme.

L: linear effect of inclusion level of hempseed, Q: quadratic effect of inclusion level of hempseed.

**Table 7:** Influence of hempseed inclusion and enzyme supplementation on ileum morphology in broilers at 28 d of age.

Treatment	VH (µm)	CD (µm)	VW (µm)	GN (100 µm)	VH:CD (µm)	VSA (mm^2^)
H0E0	465.16	231.10	56.16	13.00	2.036	0.336
H0E1	512.53	231.27	72.95	13.17	2.233	0.371
H8E0	495.00	235.81	63.61	14.00	2.103	0.367
H8E1	524.28	229.08	70.28	14.17	2.310	0.377
H16E0	518.98	205.56	83.36	14.83	2.530	0.334
H16E1	566.96	212.10	82.31	14.33	2.686	0.379
H24E0	549.65	205.42	78.53	14.50	2.716	0.353
H24E1	609.43	229.22	84.50	14.17	2.673	0.439
SEM	13.34	8.49	4.67	0.54	0.106	0.017
Interaction (Hempseed × Enzyme)						
p-value	0.722	0.327	0.311	0.89	0.625	0.202
Main effects						
Inclusion level of hempseed						
H0	488.85^c^	231.18^ab^	64.55^b^	13.08^b^	2.135^b^	0.354
H8	509.64^bc^	232.45^a^	66.95^b^	14.08^b^	2.206^b^	0.372
H16	542.97^b^	208.83^b^	82.84^a^	14.58^a^	2.608^a^	0.357
H24	579.54^a^	217.31^ab^	81.51^a^	14.33^ab^	2.693^a^	0.396
SEM	9.43	6.00	3.30	0.38	0.075	0.012
p-value	<0.0001	0.021	0.0002	0.048	<0.0001	0.079
Enzyme						
E0	507.20^b^	219.47	70.42^b^	14.08	2.345	0.348^b^
E1	553.30^a^	225.41	77.51^a^	13.96	2.475	0.391^a^
SEM	6.67	4.24	2.33	0.27	0.053	0.008
p-value	<0.0001	0.328	0.038	0.74	0.091	0.001
Orthogonal polynomial contrast						
L	0.0001	0.009	0.0004	0.037	<0.0001	0.804
Q	0.975	0.773	0.191	0.227	0.605	0.703

VH: villus height, CD: crypt depth, VW: villus width, GN: goblet cell count, VH:CD: villus height/crypt depth, VSA: villus surface area.

Means in the same column with different superscripts (^abc^) are significantly different.

SEM: pooled standard error of the mean.

H0: 0% hempseed, H8: 8% hempseed, H16: 16% hempseed, H24: 24% hempseed. E0: without enzyme, E1: 0.104 g/kg enzyme.

L: linear effect of inclusion level of hempseed, Q: quadratic effect of inclusion level of hempseed.

Hempseed inclusion significantly affected most morphometric characteristics in the duodenum, jejunum, and ileum. In the duodenum, hempseed affected VH, CD, VW, GN, VH:CD, and VSA (P ≤ 0.009). In the jejunum, hempseed affected VH, CD, GN, VSA, and VH:CD (P ≤ 0.027), whereas VW was not affected. In the ileum, hempseed affected VH, CD, VW, GN, and VH:CD (P ≤ 0.048), while VSA was not affected. Significant linear responses to increasing hempseed inclusion were observed for several morphometric traits across intestinal segments except VW in duodenum and jejunum (P = 0.55 and P = 0.436, respectively) and VSA in ileum (P = 0.804), whereas quadratic responses were less consistent.

MC supplementation affected duodenal VH, CD, and VH:CD (P ≤ 0.028), as well as jejunal CD (P = 0.001), but did not significantly affect the other measured traits in these segments. In the ileum, MC supplementation significantly affected the VH, VW, and VSA (P ≤ 0.038).

### Apparent ileal digestibility

The effects of hempseed inclusion and MC supplementation on AID at 28 d are presented in Table 8. Significant H × MC interactions were identified for AID of EE, CP, ADF, and GE (P ≤ 0.030), whereas no interaction was observed for AID of DM, OM, or NDF (P > 0.05). Mean values declined from 0% to 24% hempseed when AID of DM was measured at different percentages of hempseed inclusion. In addition, the linear response was detected as significant (P = 0.005). In contrast, MC supplementation increased AID of DM compared with the unsupplemented diets (P = 0.001). Hempseed inclusion (P = 0.042) and MC supplementation (P < 0.001) affected AID of OM, and the highest main effect of hempseed was observed at 8% inclusion. For NDF, hempseed inclusion significantly affected AID (P < 0.001), with values decreasing from 0% to 24% hempseed. MC supplementation also affected AID of NDF digestibility (P < 0.004). Significant increases in AID of EE were observed across the H × MC treatments, with an increasing trend from H0E0 to H24E0. The corresponding hempseed main-effect means increased as hempseed inclusion increased from 0% to 24% (P < 0.001), with significant linear (P < 0.001), whereas the quadratic response was not significant (P = 0.12). For AID of CP, treatment means increased from H0E0 to H24E1. The hempseed main effect increased AID of CP as hempseed inclusion increased from 0% to 24% (P < 0.001), with significant linear (P < 0.001) and quadratic (P = 0.01) responses. The highest treatment means were observed in H8E0 and the lowest in H24E0 when AID of ADF was measured. Hempseed inclusion reduced AID of ADF (P < 0.001), decreasing response from 0% to 24% hempseed. The linear response was significant (P = 0.001). The results showed that AID of GE ranged from a minimum at H0E0 to a maximum at H8E1. Hempseed inclusion, as a main effect, affected AID of GE (P < 0.001), with an increasing response from 0% to 8%, after which values remained similar at 16 and 24% inclusion. MC supplementation also significantly affected AID of GE (P < 0.001).

**Table 8:** Influence of hempseed inclusion and enzyme supplementation on apparent ileal digestibility in broilers at 28 d of age.

Treatment	DM (%)	OM (%)	EE (%)	CP (%)	ADF (%)	NDF (%)	GE (%)
H0E0	89.30	45.92	71.31^d^	50.90^c^	38.31^ab^	56.90	40.32^c^
H0E1	90.49	49.14	75.84^cd^	56.11^b^	39.76^a^	58.83	47.88^b^
H8E0	89.08	47.80	77.20^c^	54.91^b^	40.81^a^	55.79	47.05^b^
H8E1	90.28	50.96	76.52^c^	56.15^b^	38.01^ab^	58.91	51.69^a^
H16E0	88.47	47.32	84.82^b^	56.54^b^	34.98^bc^	51.66	49.01^ab^
H16E1	88.54	49.45	81.60^bc^	57.52^ab^	35.22^bc^	52.04	48.62^b^
H24E0	87.09	47.44	88.44^a^	56.71^ab^	34.47^c^	47.71	48.40^b^
H24E1	87.50	48.35	84.39^b^	59.37^a^	35.01^bc^	51.21	48.71^ab^
SEM	0.25	0.61	0.64	0.54	0.72	0.76	0.68
Interaction (Hempseed × Enzyme)							
p-value	0.09	0.23	<0.001	0.005	0.03	0.19	<0.001
Main effects							
Inclusion level of hempseed							
H0	89.89^a^	47.53^b^	73.57^d^	53.51^c^	39.04^a^	57.87^a^	44.10^b^
H8	89.68^a^	49.38^a^	76.86^c^	55.53^b^	39.41^a^	57.36^a^	49.35^a^
H16	88.50^b^	48.38^ab^	83.21^b^	57.03^ab^	35.11^b^	51.85^b^	48.81^a^
H24	87.30^c^	47.89^ab^	86.41^a^	58.04^a^	34.74^b^	49.46^c^	48.55^a^
SEM	0.18	0.43	0.45	0.38	0.51	0.54	0.48
p-value	<0.001	0.042	<0.001	<0.001	<0.001	<0.001	<0.001
Enzyme							
E0	88.49^b^	47.12^b^	80.44	54.76^b^	37.14	53.01^b^	46.18^b^
E1	89.20^a^	49.47^a^	79.59	57.29^a^	37.01	55.25^a^	49.22^a^
SEM	0.12	0.30	0.32	0.27	0.42	0.38	0.34
p-value	0.001	<0.001	0.07	<0.001	0.78	0.004	<0.001
Orthogonal polynomial contrast							
L	0.005	0.19	<0.001	<0.001	0.001	<0.001	<0.001
Q	0.07	0.21	0.12	0.01	0.05	0.005	0.002

DM: dry matter, OM: organic matter, EE: ether extract, CP: crude protein, ADF: acid detergent fiber, NDF: neutral detergent fiber, GE: Gross energy.

Means in the same column with different superscripts (^abc^) are significantly different.

SEM: pooled standard error of the mean.

H0: 0% hempseed, H8: 8% hempseed, H16: 16% hempseed, H24: 24% hempseed. E0: without enzyme, E1: 0.104 g/kg enzyme.

L: linear effect of inclusion level of hempseed, Q: quadratic effect of inclusion level of hempseed.

## Discussion

In the present study, hempseed inclusion improved BW, BWG, and FCR, while MC supplementation independently improved BWG and FCR. The increase in BW with increasing hempseed inclusion showed both linear and quadratic responses, whereas BWG was affected by hempseed inclusion without significant linear or quadratic responses. The improvement in BW and BWG may be partially associated with the modifications in AID observed in the present study, particularly for CP, EE, and GE. A comparable positive effect on growth performance was reported by Tufarelli et al. (2023) with hempseed cake, attributed to improved intestinal health and nutrient absorption. However, hempseed cake is a by-product of oil extraction and differs considerably from whole hempseed in fat, protein, and fiber contents (whole hempseed: CP=24.7, CF=29.6; hempseed cake: CP=31.3, CF=31.5). Hence, the results are not directly comparable, and the similar responses may reflect diverse nutritional mechanisms. Previous reports indicate that carbohydrase supplementation can improve feed efficiency by enhancing the utilization of nutrients in diets containing NSP-rich ingredients (Rebolé et al., 2010; Cozannet et al., 2017; Mahmoodtabar et al., 2026), which is consistent with improved BWG and FCR by MC supplementation in our report. Although the H16E1 treatment exhibited the highest numerical BWG and the most favorable FCR, the H × MC interaction was not significant for growth performance. Therefore, these numerical differences do not provide evidence of a synergistic or combined effect between hempseed and MC. The improvements observed with hempseed inclusion and MC supplementation may instead reflect their respective effects on nutrient utilization and intestinal characteristics. In the present study, higher hempseed inclusion was accompanied by changes in nutrient digestibility and selected ileal bacterial populations, while changes in intestinal morphology were also observed. This interpretation matches findings from Rebolé et al. (2010), and Cozannet et al. (2017) Who showed that enzyme complexes help improve feed efficiency by making it easier to digest ingredients that are rich in NSPs. Earlier studies have pointed out that changes in the structure of the intestine and shifts, in gut bacteria might be linked to how nutrients are used and how well broilers grow (Tufarelli et al., 2023; Incharoen et al., 2025). Since the current study did not directly test these biological processes as cause-and-effect pathways these connections should be seen as potential explanations, not proven mechanisms. The significant linear response of FCR to increasing hempseed inclusion, together with the absence of a significant quadratic response, suggests a progressive improvement in feed efficiency across the tested inclusion range rather than a statistically demonstrated plateau. A comparable limitation of excessive hemp inclusion has been reported, although in that case hempseed oil was the test ingredient (Kanbur, 2022) and its composition is not equivalent to that of whole hempseed, so the comparison is restricted to the direction of the response at the highest inclusion level.

The response of AID to hempseed inclusion was nutrient-fraction specific rather than uniform (Table 8). As dietary hempseed inclusion increased, AID of CP, EE, and GE increased, whereas AID of DM, ADF, and NDF decreased (P < 0.001 for each main effect). AID of OM was highest at 8% inclusion, with no linear trend. This pattern is consistent with the findings of Shahtalab et al. (2026), who reported higher AID of CP, EE, and GE in broilers fed 16% hempseed in place of soybean meal than in those fed a corn–soybean control diet, alongside a decrease in AID of NDF and no significant effect on AID of ADF; in the present study, in contrast, the decrease in AID of ADF was significant. The agreement between the two datasets suggests that the responses to whole hempseed are associated with the nutrient composition of the ingredient (Table 2) rather than with the conditions of the present experiment. As previously reported, birds fed hempseed alone had higher CP, EE, and GE than the control, which supports our observations (Khan et al., 2010; Sana et al., 2024; Tufarelli et al., 2023).

Despite the diets being formulated to be isonitrogenous and isocaloric, the higher AID of CP cannot be attributed to a greater dietary protein supply. Replacing soybean meal with hempseed changes both the protein source and the matrix in which the protein is embedded. Plant protein sources differ in protein structure, amino acid distribution, and association with cell-wall components, which can affect protein accessibility to digestive enzymes, and the protein digestibility of hempseed products has been reported to vary with the seed fraction and hull content (House et al., 2010). In addition, the apparent coefficients for CP and DM are influenced by endogenous nitrogen and organic matter losses (Ravindran et al., 1999) and are influenced by the intestinal microbiota, which affects the microbial and endogenous protein fractions in ileal digesta (Apajalahti and Vienola, 2016). The present experiment does not allow the relative contributions of these mechanisms to be distinguished.

The increase in AID of EE is consistent with the change in the dietary lipid source. As hempseed inclusion increased, supplemental soybean oil was progressively replaced by the lipid naturally present in hempseed, which differs from soybean oil in fatty acid composition, and the total amount of lipid supplied also increased, since whole hempseed contains 30.5% ether extract (Table 2). The response therefore reflects a change in the composition of dietary fat that cannot be separated from the accompanying increase in the amount of fat supplied, and it cannot be attributed to specific fatty acid classes because the fatty acid composition of the diets and of ileal digesta was not determined.

In contrast, the decreases in AID of DM, ADF, and NDF are consistent with the increasing contribution of the structural fiber fraction of whole hempseed (Table 2). As hempseed inclusion increased, and higher dietary fiber is generally associated with lower nutrient digestibility in poultry (Jha and Mishra, 2021). Broilers also lack the endogenous enzymes needed to hydrolyze structural plant cell-wall carbohydrates, which are degraded mainly by microbial fermentation (Choct, 2015). Part of the decline in these coefficients may therefore be attributable to changes in dietary composition rather than to a reduction in the digestive capacity of the bird. The concurrent improvement in the utilization of CP, EE, and GE and the decline in fiber digestibility is not contradictory; rather, they reflect the different digestibilities of the individual nutrient fractions.

Multicarbohydrase supplementation increased AID of DM, OM, CP, NDF, and GE as main effects but had no significant effect on EE or ADF. These findings are consistent with the hydrolytic activities of xylanase and β-glucanase against arabinoxylans and β-glucans, which may increase nutrient accessibility within the cell-wall matrix and reduce the viscosity associated with soluble NSP. Similar improvements in nutrient and energy utilization and reductions in digesta viscosity following xylanase and β-glucanase supplementation have been reported by Kim et al. (2025). Furthermore, NSP degradation along the gastrointestinal tract has been investigated in broilers receiving xylanase-based and broader NSP-degrading enzyme preparations by Morgan et al. (2022).

Significant H × MC interactions were detected for AID of EE, CP, ADF, and GE (P ≤ 0.030), but not for DM, OM, or NDF (P > 0.05). Thus, the effects of MC on DM, OM, and NDF did not vary significantly with hempseed inclusion level, whereas a single overall enzyme effect could not adequately describe the responses of EE, CP, ADF, and GE across all inclusion levels. These interactions may reflect changes in the amount and type of enzyme-responsive substrates as the dietary matrix changed, although this interpretation was not directly tested. Further investigation involving the characterization of dietary NSP fractions, together with measurement of digesta viscosity and of endogenous losses, would help clarify the mechanisms underlying these responses.

Hempseed inclusion reduced total aerobic bacteria and coliform counts and increased *Lactobacillus* spp. as main effects (P ≤ 0.009), with significant linear and quadratic responses for all three populations (P ≤ 0.048; Table 4). Multicarbohydrase supplementation produced changes in the same direction but of smaller magnitude (P ≤ 0.032). These findings are partly consistent with previous studies reporting reduced coliform counts and increased lactobacilli in broilers fed hempseed (Vispute et al., 2019), although that study used hempseed at 0.2–0.3% inclusion and sampled cecal and jejunal digesta, so the comparison is limited to these two populations. In broilers given 16% raw hempseed in place of soybean meal from day 8–28, ileal *E. coli* counts were lower with hempseed alone, whereas lactobacilli were reduced only in the treatment that also received a carbohydrase blend and butyric acid (Shahtalab et al., 2026); in birds given hempseed cake, total bacterial and *E. coli* counts were higher with the unheated cake and lower with the autoclaved form, while *Lactobacillus acidophilus* was increased by the heated cake (Darmawan and Ozturk, 2025). The form in which the hemp product is supplied, the processing it receives and the additives given with it therefore all appear to influence the bacterial response.

The effects of hempseed may reflect changes in dietary fiber, its constituent NSPs, and other seed constituents; however, their individual contributions cannot be distinguished because these components changed simultaneously. MC may likewise alter the availability of carbohydrate substrates through NSP hydrolysis (Morgan et al., 2022), and reductions in ileal pH and digesta viscosity have been reported with xylanase and β-glucanase supplementation (Kim et al., 2025), whereas an exogenous multi-enzyme with phytase left ileal and cecal bacterial populations unchanged (Kim et al., 2021). Significant H × MC interactions were observed for total aerobic bacteria (P < 0.001) and *Lactobacillus* spp. (P = 0.009), but not for coliforms (P = 0.335), indicating that the effects of MC on the first two populations depended on hempseed inclusion level, the greatest numerical reduction in total aerobic bacteria occurring at 8% hempseed. Several limitations should be considered when interpreting the microbial results. Bacterial enumeration was culture-based and restricted to ileal digesta, so these findings reflect changes in selected culturable bacterial populations in the ileum rather than in the overall intestinal microbiota. In addition, the caeca, rather than the ileum, are the principal site of microbial fermentation in chickens (Rinttilä and Apajalahti, 2013), so the data do not indicate changes in fermentative activity. Therefore, further investigation of bacterial populations at the species level and assessment of the cecal microbiota may help clarify the responses to hempseed and MC. The specific contributions of individual hempseed components and the mechanisms underlying the observed microbial responses remain to be elucidated.

Segment-specific intestinal morphology was modified by dietary hempseed and MC supplementation, consistent with the observed changes in nutrient digestibility and microbial profile. In our study, higher hempseed inclusion (particularly 16 and 24%) increased the VH and VH:CD ratio in the duodenum and jejunum, while CD tended to decrease or remain unchanged in most segments. Previous studies have reported that increases in VH, VSA, and VH:CD ratio were achieved when broilers were fed 10% hempseed cake, indicating improved absorptive capacity and gut health (Tufarelli et al., 2023). This is also supported by Shahtalab et al. (2026), who found that replacing 16% of soybean with hempseed in broiler feed could decrease CD and increase the VH:CD ratio in the jejunum. Mechanistically, the improvements in digestibility and microbial balance may help explain the observed enhancement in villus morphology. Improved CP, EE, and GE digestibility may reduce the amount of undigested substrate reaching the distal ileum, which could lower fermentative pressure on the mucosa in that segment (Ravindran and Abdollahi, 2021; Ducatelle et al., 2023). This explanation is most plausible for the ileum, whereas the duodenal and jejunal responses are less likely to be explained by distal substrate flow. In these proximal segments, other factors may be involved, such as reduced digesta viscosity from enzymatic NSP degradation and changes in the luminal nutrient supply. A higher VH:CD ratio is commonly interpreted as an indicator of intestinal morphology, while greater villus height may reflect changes in the mucosal structure (Awad et al., 2009). The observed morphological changes are structural responses to dietary treatments. Likewise, increased villus height is associated with a larger absorptive surface area available for nutrient uptake, which may partially explain the improvements observed in nutrient digestibility and growth performance. Previous studies have reported associations between intestinal bacterial populations and mucosal morphology in broilers (Šefcová et al., 2023; Hartoyo et al., 2023). Therefore, the observed bacterial and morphological responses are concurrent changes associated with dietary treatments. Short-chain fatty acids produced by certain intestinal bacteria have been associated with epithelial integrity and intestinal morphology in poultry (Bawish et al., 2023). However, short-chain fatty acids and epithelial integrity were not directly assessed in the present study; therefore, this mechanism remains a possible explanation rather than a demonstrated pathway. Additional support comes from Kanbur (2022), who observed alterations in intestinal structure in broilers fed hempseed oil, with improvements in mucosal integrity linked to hemp’s omega-3 fatty acids and antioxidant components. Multicarbohydrase supplementation was associated with changes in several intestinal morphometric parameters. In the present study, MC supplementation increased VH and VH:CD and reduced CD in the duodenum, reduced CD in the jejunum, and increased VH, VW, and VSA in the ileum. These findings indicate changes in intestinal morphology, although their functional implications cannot be established from morphometric measurements alone. Similar increases in VH:CD and decreases in CD with xylanase supplementation were reported by González-Ortiz et al. (2019) in the ileum. Overall, the findings show that dietary hempseed inclusion and MC supplementation were associated with changes in intestinal morphometric parameters. These structural responses provide useful information about the intestinal effects and provide new insights into how these dietary strategies interact to improve nutrient bioavailability, gut health, and overall growth performance, but further functional measurements would be needed to determine whether they translate into improved intestinal absorptive function. In the duodenum, the H16E1 treatment resulted in significantly greater VH and VH:CD ratio than the other treatments. This response suggests that the H × MC interaction on duodenal morphology was not uniform across treatment combinations. In particular, the pronounced response in H16E1 indicates that the combination of 16% hempseed and MC was associated with distinct structural changes in the duodenal mucosa.

## Conclusion

Under the conditions of this study (d 8–28), dietary hempseed improved BW, BWG, and FCR and modified ileal nutrient digestibility, intestinal morphometric characteristics, and the counts of selected ileal bacteria. Multicarbohydrase independently improved BWG, FCR, and the AID of several nutrient fractions. Significant H × MC interactions for selected digestibility and morphometric traits indicate that some responses to MC varied with hempseed inclusion level, whereas no interaction was detected for growth performance. The morphometric changes do not by themselves establish improved absorptive function. Overall, hempseed shows potential as an alternative ingredient in broiler diets, and MC can improve the utilization of selected nutrients. Further studies are needed to determine the optimal hempseed level and whether the intestinal morphological changes translate into measurable improvements in intestinal function.

## Author Contribution

We would like to confirm that an Author Contribution Statement in the requested format was already provided with our submission. It does not use CRediT terminology, and it describes the role of each author in line with the four authorship requirements specified by the journal: substantial contributions to the work, drafting or critical review of the manuscript, approval of the final version, and accountability for all aspects of the work.

## Disclosures

The authors declare that they have no known competing financial interests or personal relationships that could have appeared to influence the work reported in this paper.

## References

[bib0001] Adeola O., Cowieson A.J. (2011). Board-invited review: opportunities and challenges in using exogenous enzymes to improve nonruminant animal production. J. Anim. Sci..

[bib0002] AOAC (2000). Official Methods of Analysis.

[bib0003] Apajalahti J., Vienola K. (2016). Interaction between chicken intestinal microbiota and protein digestion. Anim. Feed Sci. Technol..

[bib0004] Aviagen (2022). https://aviagen.com.

[bib0005] Awad W.A., Ghareeb K., Abdel-Raheem S., Böhm J. (2009). Effects of dietary inclusion of probiotic and synbiotic on growth performance, organ weights, and intestinal histomorphology of broiler chickens. Poult. Sci..

[bib0006] Bawish B.M., Zahran M.F.S., Ismael E., Kamel S., Ahmed Y.H., Hamza D., Fahmy K.N.E. (2023). Impact of buffered sodium butyrate as a partial or total dietary alternative to lincomycin on performance, IGF-1 and TLR4 genes expression, serum indices, intestinal histomorphometry, clostridia, and litter hygiene of broiler chickens. Acta Vet. Scand..

[bib0007] Callaway J.C. (2004). Hempseed as a nutritional resource: an overview. Euphytica.

[bib0008] Choct M. (2015). Feed non-starch polysaccharides for monogastric animals: classification and function. Anim. Prod. Sci..

[bib0009] Cozannet P., Kidd M.T., Neto R.M., Geraert P.A. (2017). Next-generation non-starch polysaccharide-degrading, multi-carbohydrase complex rich in xylanase and arabinofuranosidase to enhance broiler feed digestibility. Poult. Sci..

[bib0010] Darmawan A., Ozturk E. (2025). Effect of dietary heated hemp seed cake and phytase as soybean meal substitution on broiler chicken performance, carcass yield, visceral organ weight, intestinal health, and serum biochemical parameters. Trop. Anim. Health Prod..

[bib0011] Dhaver P., Pletschke B., Sithole B., Govinden R. (2022). Optimization, purification, and characterization of xylanase production by a newly isolated Trichoderma harzianum strain by a two-step statistical experimental design strategy. Sci. Rep..

[bib0012] Ducatelle R., Goossens E., Eeckhaut V., Van Immerseel F. (2023). Poultry gut health and beyond. Anim. Nutr..

[bib0013] González-Ortiz G., Dos Santos T.T., Vienola K., Vartiainen S., Apajalahti J., Bedford M.R. (2019). Response of broiler chickens to xylanase and butyrate supplementation. Poult. Sci..

[bib0014] Hartoyo B., Prihambodo T.R., Wahyuningsih S.R., Suhartati F.M., Bata M., Rimbawanto E.A. (2023). *Lactobacillus* as growth promoter: a meta-analysis of performance, histology and microbiota on broiler tract digestive. Adv. Anim. Vet. Sci..

[bib0015] Hetland H., Choct M., Svihus B. (2004). Role of insoluble non-starch polysaccharides in poultry nutrition. World's Poult. Sci. J..

[bib0016] House J.D., Neufeld J., Leson G. (2010). Evaluating the quality of protein from hemp seed (Cannabis sativa L.) products through the use of the protein digestibility-corrected amino acid score method. J. Agric. Food Chem..

[bib0017] Incharoen T., Nopparatmaitree M., Kongkeaw A., Soisuwan K., Likittrakulwong W., Thongnum A., Loor J.J. (2025). Dietary micronized hemp fiber enhances in vitro nutrient digestibility and cecal fermentation, antioxidant enzyme, lysosomal activity, and productivity in finisher broilers reared under thermal stress. Front. Anim. Sci..

[bib0018] Jha R., Mishra P. (2021). Dietary fiber in poultry nutrition and their effects on nutrient utilization, performance, gut health, and on the environment: a review. J. Anim. Sci. Biotechnol..

[bib0019] Kanbur G. (2022). Growth-depressing effect of dietary hempseed oil on broiler performance in the starting period and alterations in meat oxidation, serum parameters and abdominal fatty acids. Anim. Sci. Pap. Rep..

[bib0020] Khan R.U., Durrani F.R., Chand N., Anwar H. (2010). Influence of feed supplementation with Cannabis sativa on quality of broilers carcass. Pak. Vet. J..

[bib0021] Kim E., Choct M., Fickler A., Pasquali G.A., Hall L., Crowley T.M., Sharma N.K. (2025). Supplementation of β-mannanase alone or in combination with xylanase and β-glucanase enhanced growth performance, non-starch polysaccharide degradation, and gastrointestinal environment of broilers offered wheat-based diets. Anim. Nut..

[bib0040] Kim MJ., Ingale S.L., Hosseindoust A., Choi Y., Kim K., Chae B. (2021). Synergistic effect of exogenous multi-enzyme and phytase on growth performance, nutrients digestibility, blood metabolites, intestinal microflora and morphology in broilers fed corn-wheat-soybean meal diets. Anim. Biosci..

[bib0022] König J., Grasser R., Pikor H., Vogel K. (2002). Determination of xylanase, β-glucanase, and cellulase activity. Anal. Bioanal. Chem..

[bib0023] Mahmoodtabar A., Shariatmadari F., Karimi Torshizi M.A., Ahmadi H. (2026). Evaluating raw and heat-treated hempseed (Cannabis sativa L) with enzyme supplementation for broiler chicken. JAST.

[bib0024] Morgan N., Bhuiyan M.M., Hopcroft R. (2022). Non-starch polysaccharide degradation in the gastrointestinal tract of broiler chickens fed commercial-type diets supplemented with either a single dose of xylanase, a double dose of xylanase, or a cocktail of non-starch polysaccharide-degrading enzymes. Poult. Sci..

[bib0025] Ravindran V., Abdollahi M.R. (2021). Nutrition and digestive physiology of the broiler chick: state of the art and outlook. Animals.

[bib0026] Ravindran V., Cabahug S., Ravindran G., Bryden W.L. (1999). Influence of microbial phytase on apparent ileal amino acid digestibility of feedstuffs for broilers. Poult. Sci..

[bib0027] Rebolé A., Ortiz L.T., Rodríguez M., Alzueta C., Treviño J., Velasco S. (2010). Effects of inulin and enzyme complex, individually or in combination, on growth performance, intestinal microflora, cecal fermentation characteristics, and jejunal histomorphology in broiler chickens fed a wheat-and barley-based diet. Poult. Sci..

[bib0028] Rinttilä T., Apajalahti J. (2013). Intestinal microbiota and metabolites—implications for broiler chicken health and performance. J. Appl. Poult. Res..

[bib0029] Saleh A.A., Kirrella A.A., Abdo S.E., Mousa M.M., Badwi N.A., Ebeid T.A., Mohamed M.A. (2019). Effects of dietary xylanase and arabinofuranosidase combination on the growth performance, lipid peroxidation, blood constituents, and immune response of broilers fed low-energy diets. Animals.

[bib0030] Sana B., Chand N., Naz S., Alhidary I.A. (2024). Effects of hempseed (Cannabis sativa L.) in diet on growth, gut health, and immunity in broilers. Vet. Q..

[bib0031] Šefcová M.A., Ortega-Paredes D., Larrea-Álvarez C.M., Mina I., Guapás V., Ayala-Velasteguí D., Larrea-Álvarez M. (2023). Effects of *Lactobacillus* fermentum administration on intestinal morphometry and antibody serum levels in Salmonella-Infantis-challenged chickens. Microorganisms.

[bib0032] Serrapica F., Lambiase C., Masucci F., Braghieri A., Lorenzo J.M., Di Francia A. (2025). Feed-grade hempseed as alternative protein source in organic broilers diet: growth performance, carcass characteristics and meat quality. J. Agric. Food Res..

[bib0033] Shahtalab A., Shariatmadari F., Torshizi M.A.K., Ahmadi H. (2026). Hempseed-based diets supplemented with carbohydrase and butyric acid for broiler chickens. Poult. Sci..

[bib0034] Shariatmadari F. (2023). Emergence of hemp as feed for poultry. World's Poult. Sci. J..

[bib0035] Short F.J., Gorton P., Wiseman J., Boorman K.N. (1996). Determination of titanium dioxide added as an inert marker in chicken digestibility studies. Anim. Feed Sci. Technol..

[bib0036] Tufarelli V., Losacco C., Tedone L., Passantino L., Tarricone S., Laudadio V., Colonna M.A. (2023). Hemp seed (Cannabis sativa L.) cake as sustainable dietary additive in slow-growing broilers: effects on performance, meat quality, oxidative stability and gut health. Vet. Q..

[bib0037] Van Soest P.V., Robertson J.B., Lewis B.A. (1991). Methods for dietary fiber, neutral detergent fiber, and nonstarch polysaccharides in relation to animal nutrition. J. Dairy Sci..

[bib0038] Vispute M.M., Sharma D., Mandal A.B., Rokade J.J., Tyagi P.K., Yadav A.S. (2019). Effect of dietary supplementation of hemp (Cannabis sativa) and dill seed (Anethum graveolens) on performance, serum biochemicals and gut health of broiler chickens. J. Anim. Physiol. Anim. Nutr..

[bib0039] Wang C., Yuan T., Yang J., Zheng W., Wu Q., Zhu K., Zhu Y. (2022). Responses of combined non-starch polysaccharide enzymes and protease on growth performance, meat quality, and nutrient digestibility of yellow-feathered broilers fed with diets with different crude protein levels. Front. Vet. Sci..

